# 3D bioprinting and the revolution in experimental cancer model systems—A review of developing new models and experiences with *in vitro* 3D bioprinted breast cancer tissue-mimetic structures

**DOI:** 10.3389/pore.2023.1610996

**Published:** 2023-02-09

**Authors:** Dániel Sztankovics, Dorottya Moldvai, Gábor Petővári, Rebeka Gelencsér, Ildikó Krencz, Regina Raffay, Titanilla Dankó, Anna Sebestyén

**Affiliations:** Department of Pathology and Experimental Cancer Research, Semmelweis University, Budapest, Hungary

**Keywords:** cancer, breast cancer, 3D bioprinting, disease models, biofabrication

## Abstract

Growing evidence propagates those alternative technologies (relevant human cell-based—e.g., organ-on-chips or biofabricated models—or artificial intelligence-combined technologies) that could help *in vitro* test and predict human response and toxicity in medical research more accurately. *In vitro* disease model developments have great efforts to create and serve the need of reducing and replacing animal experiments and establishing human cell-based *in vitro* test systems for research use, innovations, and drug tests. We need human cell-based test systems for disease models and experimental cancer research; therefore, *in vitro* three-dimensional (3D) models have a renaissance, and the rediscovery and development of these technologies are growing ever faster. This recent paper summarises the early history of cell biology/cellular pathology, cell-, tissue culturing, and cancer research models. In addition, we highlight the results of the increasing use of 3D model systems and the 3D bioprinted/biofabricated model developments. Moreover, we present our newly established 3D bioprinted luminal B type breast cancer model system, and the advantages of *in vitro* 3D models, especially the bioprinted ones. Based on our results and the reviewed developments of *in vitro* breast cancer models, the heterogeneity and the real *in vivo* situation of cancer tissues can be represented better by using 3D bioprinted, biofabricated models. However, standardising the 3D bioprinting methods is necessary for future applications in different high-throughput drug tests and patient-derived tumour models. Applying these standardised new models can lead to the point that cancer drug developments will be more successful, efficient, and consequently cost-effective in the near future.

## Introduction

### “ALL models are wrong but some are useful.”

This April, the European Union (EU) prohibited selling new cosmetic products tested on animals without any exemptions (1). Accordingly, it is forbidden to place a new product on the market that contains even one new ingredient tested on animals in or out of the EU, even if the final product was not tested on animals (2). It is also well-known that pharmaceutical drug development is extremely time-consuming and expensive; moreover, about 90% of drugs fail after preclinical animal testing in human safety and efficacy trials. Additionally, in patients, ∼1/6 newly marketed drugs are withdrawn or discontinued due to serious adverse effects (e.g., hepatic, cardiovascular, hematologic, neurologic, and carcinogenic) (3). Potentially beneficial drugs can also fail and never be placed on the market if these are categorised as toxic or ineffective during preselection based on animal tests (e.g., in recent conditions, aspirin could fail regarding its toxicity test results on rats and rhesus monkey embryos). Thus, certain patients could not benefit or even be omitted due to the limitations of animal models (4). Both U.S. Food and Drug Administration (FDA) and European Medicine Agency (EMA) modernisation acts aim to replace and decrease animal testing. Therefore, the bioengineering research area has rapidly improved in the last decade. Moreover, *in vitro* human model systems have undergone enormous developments nowadays. In the last pandemic situation, the development of COVID vaccines showed that faster and more efficient technologies are necessary for crisis and medical developments, and we have to leap over animal experiments and use clinical tests as fast as possible (5). Growing evidence propagates those alternative technologies (relevant human cell-based—e.g., organ-on-chips or biofabricated models—or artificial intelligence-combined technologies) could help test and predict human response and toxicity in medical research more accurately (6, 7).

*In vitro* disease model developments have great efforts to create and serve the need of reducing, and replacing animal experiments and establishing human cell-based *in vitro* test systems for research use, innovations, and drug tests. We know and accept the quote: “ALL models are wrong but some are useful”—(George E.O. Box, one of the greatest statisticians of the 20th century). We need human cell-based test systems for disease models and experimental cancer research; therefore, *in vitro* three-dimensional (3D) models have a renaissance, and the development of these technologies is growing ever faster.

### The early history of cell biology/cellular pathology, cell-, tissue culturing, and cancer research models

**FIGURE 1:**
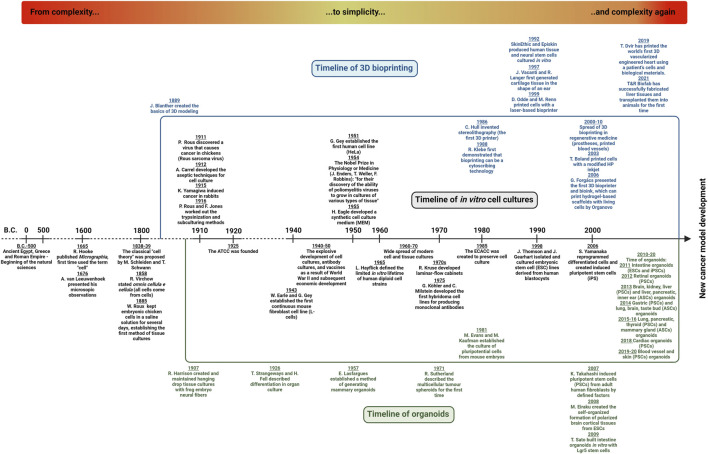
Timeline of cell biology and experimental cancer models—From complexity…to simplicity…and complexity again. The three research areas—*in vitro* cell- and tissue-culturing, organoid technology and 3D bioprinting—are developing, and their co-evolution with cancer research supports the establishment of new cancer models. A detailed explanation can be found in the text.

Aristotelian doctrine about spontaneous generation describes those non-living substances (water, stones, and salts) that have some additional potential to spontaneously create complex systems and organisms. Regarding these explanations, insects, and flies are developed from mud and inorganic matter (8). The revolution of cell biology and the birth of cellular biology would not have been possible without one great invention—the microscope, after the invention of convex lenses by *Janssen* and the telescopes created by *Galilei*. Among many other early medical descriptions (9) *Hook* and *van Leeuwenhoek* made their unusual discovery of the invisible microscopic world (life) at the beginning of the 17th century (10). *van Leeuwenhoek* handcrafted lenses and constructed microscopes. In 1665, *Hook* published his outstanding findings in *Micrographia*; he described and illustrated many biological entities as well as defined microscopic units (e.g., “cells” or “pores”). The term “cell” directly comes from this work. The spontaneous generation doctrine was experimentally disproved and laid by *Pasteur* proving that life arises from pre‐existing living organisms (it was demonstrated that microorganisms are present even in the air) (11). The developments in microscopic technologies helped to improve and even carry out more detailed studies. *Virchow*, *Schwann,* and *Kölliker* examined cells and tissues and also observed that “the elementary parts of all tissues are formed from cells” and suggested that “there is one universal principle of development for the elementary parts of organisms…and this principle is in the formation of cells” (12). Schwann and his colleagues described that the “cells arise inside and near other cells by differentiation of a homogenous primary substance” (13). *Virchow* and other scientists presented the view that cells are formed *via* the scission of pre-existing cells. Finally, in 1858, *Virchow* defined the *cell* as the fundamental unit of life, and he also created a pathogenic concept—accordingly, diseases are the results of changes in normal cells (“cells with bad behaviour”) (14). In addition, he was the one who laid the base of cellular pathology in 1863 (15) (Figure 1).

The first registered successful *in vitro* cultures were performed by *Roux*. He could maintain living cells (from chick embryos) outside the body for a few days (16). In parallel, *Loeb* could culture skin fragments from guinea pigs in agar culture (17). Then, *Harrison* developed the first, later termed hanging drop technology from small pieces of frog embryonic tissues, as well as described and introduced the aseptic method which could maintain sterile cell specimens *in vitro* for weeks (18). In New York, *Carrel* and his co-workers established cell cultures *in vitro* using embryonic and adult tissues of many species maintained in special culture media; they were able to culture their cells and tissues for several months (19). They started to work with cancer tissues and also introduced the term “tissue culture.” In 1912, the first “cell line” derived from an explanted chicken embryo heart was also established by *Carrel*. In 1938, based on his observations and studies, he published a book entitled “The Culture of Organs”. In the 1920s, the introduction of the tissue trypsinization method had a significant impact on cell culture development (20). *Rous* and his colleague were able to produce single-cell suspension from tissues, detach and subculture adherent cells obtaining homogenous cell strains which were successfully performed with this innovation.

After establishing some animal cell lines, the first human cell line (HeLa derived from cervix cancer) was established by *Gey* in 1951. In 1961, *Hayflick* inspired by *Carrel’s* observations expected that cancer cells have special cancer‐like properties (immortalised and gain limitless proliferation potential), but normal human fibroblasts have restricted growth potential (limited division capacity), and some other normal cells did not grow any longer (21). In parallel, cancer research development, tumorigenesis theories, and developing knowledge about spontaneous or induced transformation allowed viral and cell fusions, to immortalise isolated normal cells, and produce antibodies or recombinant proteins using hybridoma, selection, and molecular biology technologies (22).

In 2006, after the development and isolation of stem cells, *Yamanaka* and his colleagues described that mouse tail‐tip adult fibroblasts can be reprogramed to stem cells by the simultaneous induction of four transcription factors—Oct3/4, Sox2, Klf4, and c‐Myc. These and the developed induced pluripotent stem cell (iPSC) technologies provide the capacity to form tissues of all three germ layers for tissue cultures (23). Additionally, *Takahashi’s* pioneer works contributed to the establishment of pluripotent stem cell culturing technologies in many laboratories in the last decades. These results have great importance in medical science, transplantation, oncology, and regenerative medicine, as well.

The history of cancer model system development started at the beginning of the last century in spite of the fact that human tumours are as “old” as human life. There were several prehistorical findings, and the first written documents can be found on *Imhotep’s* papyrus with 48 described surgical cases, including some breast and other cancers (24). In the ancient ages, *Hippocrates* had many observations about malignant tumours which reminded him of the moving crabs, therefore, he named the disease *cancer*. *Celsus* highlighted the tumours’ invasive behaviour, and in the Middle Ages, *Fallopius* distinguished benign and malignant tumours, as well. Cancer epidemiology studies were started by *Ramazzini* and *Hill* (breast cancers in nuns and testicular cancers in chimney sweeps) in the 17th–18th centuries (25). However, the first Cancer Hospital was established in Reims (France) in 1779, and there were many described and treated cases, the first experimental models on tumours were performed only at the beginning of the last century with chickens and rabbits **(** *Rous*, *Yamagiwa,* and *Ichikawa*) (26). After these first models, the number of cancer research experiments increased and the fast development of cell culturing technologies began. The American Type Cell Collection (ATCC) was founded in 1925 and 60 years later, the European one (European Collection of Authenticated Cell Cultures - ECACC) was also established. These developments contributed to the discovery of the first oncogene related to the first viral carcinogenesis experiments with *Rous* sarcomas in chicken (27). Carcinogenesis-related experiments and implantation of human tumours in immunosuppressed animals were developed, however, all these models have some limitations which need to be considered (28,29,30).

For decades, researchers’ studies relied on a combination of cell culture and animal models for studying cellular mechanisms that lead to human diseases. These models have limited ability to recapitulate the complex tissue microenvironment, organ and body structures. The recognition of the cellular properties and role of tissue microenvironment have motivated the development and use of new techniques/3D cell culturing technologies/biofabrication in more complex *in vitro* models (31). However, the clinical translation ability of animal models to human disease has been questioned, in correlation with low success rates of clinical phase trials after many promising animal experiments (32). Furthermore, the high cost, the strict ethical regulations and concerns in animal testing are initiated to find less expensive, more predictive and human cell- and extracellular matrix (ECM)-based alternatives. 3D *in vitro* models are proposed to be bridges between cell cultures and *in vivo* animal models and even human trials. Early 3D culturing studies involved explanted host tissue, slice cultures and different spheroid cultures using hanging drop cultures, ultra-low attachment plates (ULA), and natural biopolymers (e.g., collagen, cell-extracted native extracellular matrix, matrigels, or polyacrylamide, agarose gels).

Regarding the increasingly well-known and renewed characteristics of various tumour tissues (33), as well as the enormously intensive therapeutic developments, their cost/benefit ratio, and many unsatisfactory clinical test results clear that new models switch to 3D technological platforms are necessary to better understand the background of the resistance mechanisms and tumour evolution. Mimicking the native cellular environment as precisely as possible is the first fundamental step to developing 3D human disease model systems (34). Recent newly developed 3D cell culturing methods (even combined with organoid or stem cell technologies) could be better and hopefully help more in drug screening in the preclinical phase and personalised treatments, as well (35).

### Going beyond standard 3D models—3D bioprinting as a new technology for disease modelling

**FIGURE 2:**
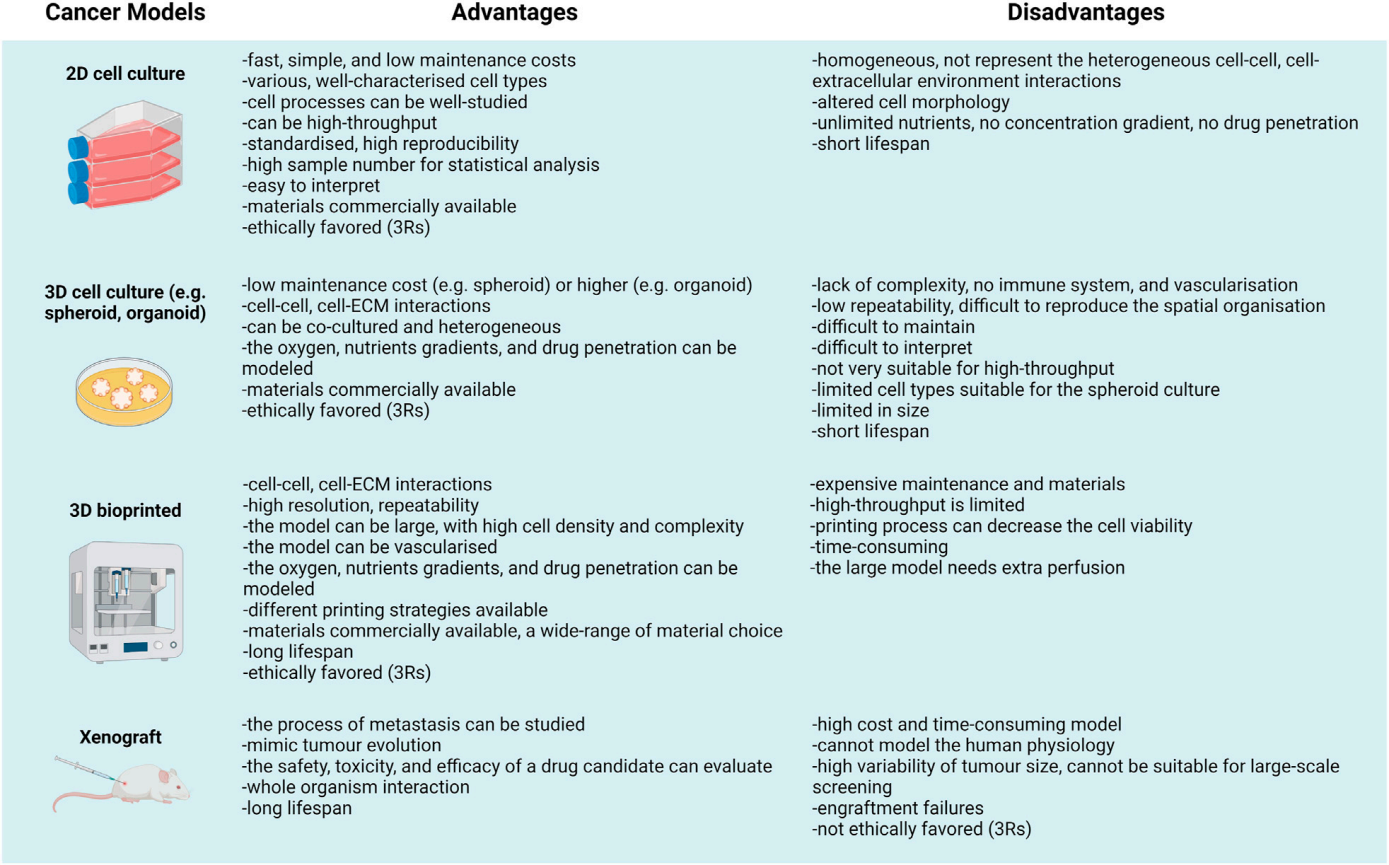
“All models are wrong but some are useful”—Advantages and disadvantages of different cancer models.

The organoid cultures re-appeared in the late 1950s (e.g., *Lasfargues* established mammary organoids), and after the emerging technologies, they have been widely applied to support pluripotent stem cell culturing and differentiation studies since the late 2010s. Regarding the results of many 3D culturing systems, the tissue-like structures of 3D cell cultures except organoids are far from *in vivo* or *in situ* environment. Additionally, there are certain *in vivo* models in which human tumours such as xenografts in SCID (severe combined immunodeficiency) mice or initiated tumorigenesis could also be investigated. Although it is getting more and more challenging to get permission to perform animal experiments; moreover, there is a need to make efforts to apply the 3R rules (replacement, reduction, refinement) as a consequence, *in vivo* studies are more and more difficult to perform. Besides, interpreting the observations made in animal experiments also poses a challenge since animal model systems could neither represent the human cellular microenvironment nor the ECM (35, 36). Comparing different options for 3D culturing and even the *in vivo* models, the advantages and disadvantages of the given experimental system have to be taken into account (Figure 2). Accordingly, the limitations of model systems have to be considered during data evaluation. 3D models are more applicable for reproducing the mechanical and biochemical characteristics, e.g., cell-cell/matrix connections, tissue stiffness, and the gradient distribution of certain factors in tumour tissues (37–39).

Among the new cutting-edge solutions, 3D bioprinted models and the continuous technological developments of tumour models could make novel opportunities for more effective pharmaceutical tests and even for the testing of personalised therapeutic alternatives (35). Combining new cell culturing technologies with applying more complex organoid cultures is expected to result in significantly better disease models. In 1992, the Episkin technique initiated the use of synthetic tissue-like cultures with the application of different cell layers mimicking the epidermis-like structures.

**A further breakthrough in tissue engineering and biofabrication is the establishment of 3D bioprinting** - Following the first stereolithographic (SLA) printer designed by *Hull* in the 1980s, the initial developmental steps of bioprinting were formed rapidly, and these coincided with the advancement of 3D printing. The primary bioprinting process, only in two-dimensional (2D), can be attributed to the research group of *Thomas Boland* (2003) (40). Soon after that, the first 3D bioprinter was also completed, which was able to print more complex multi-layered structures. It is well-known that the Hungarian-born *Gábor Forgács* and the company named Organovo had a significant role in the development of the 3D bioprinting platform (41). Furthermore, the various bioprinters that have been widespread since 2010 have established the conditions for the creation of 3D printed mini-organs, or even complete organs, with which the printing of “beating hearts”, future transplantable hearts, or other replaceable tissues can be started (42–44). In parallel with these developments, various living tissues, prostheses, and other bioprinted structures and devices have appeared. Such devices are not only being developed, but are already in use in regenerative medicine and dentistry (45, 46), and there are also considerable efforts in the development of testing active drug ingredients (organ-on-chip technology for drug toxicity tests) or drug formulation (e.g., 3D bioprinted pills) and the improvement of various disease models. Additive manufacturing (47), 3D printing and thus 3D bioprinting technology can enable the designed structure to be generated rapidly layer-by-layer by applying computer-aided design (CAD).

**Several methods** - Have been developed which can be classified based on the printing technology with cell-contained bioinks: 1) drop-based (inkjet- or laser-based bioprinting), 2) extrusion-based, and 3) digital light processing (DLP) or SLA printing. In contrast with other printing methods, the benefits of the most common extrusion-based printing technology are the choice of resolution (fibre thickness)—which of course depends on the material –, fast execution, and relative cost-effectiveness (48). Traditionally, the materials used in 3D printing for medical purposes were inert and cell-free, like plastic (49). As a result of the developments, other types of materials are now also used during 3D bioprinting as biocompatible or biodegradable materials for creating implants or soft tissue reconstruction (e.g., bone replacements, reconstructive plastic surgery) (50, 51). One of the newest areas that have developed from 3D printing is 3D bioprinting with live cells, during which so-called bioinks consisting of cells mixed in a matrix material are used for printing. Applying these, we can create a living tissue-like structure in the course of their long-term *in vitro* culturing (52). The requirements for bioinks were summarised by Groll et al. as follows: bioink is a cellular preparation that contains biologically active components and biomaterials and can be used in automated production technology (53). The primary criteria for an ideal scaffold or bioink are to provide a suitable environment for cell adhesion, proliferation, differentiation, and migration, as well as cell-matrix interactions. Tumour cells form and develop in a rather complex, multicellular-originated, heterogeneous environment which is made up of various cell types and extracellular matrix components.

**Different bioinks** - Used in the field of tumour modelling are biomaterials that consist of mixtures of hydrogels and primarily tumour cells and tumour-associated normal cells. Numerous bioinks are biomaterials that are combinations of hydrogels and cells. Bioinks can be made from naturally occurring ingredients, but synthetic versions are also available (54). It is required that the substances of bioinks have appropriate mechanical and biodegradable characteristics. For example, printability and later shape retention are important, so the bioink must meet certain mechanical conditions, e.g., easy application and a high degree of shape fidelity. It is not negligible that the materials used for printing are not allowed to cause cell death, thus these have to be biocompatible or biodegradable even after the printing process (55, 56). The most frequently used components of bioinks are the hydrogels, of which the main ingredient is water, imitating the natural cellular environment (57).

Natural hydrogels differ from synthetic ones; bioinks belonging to the former type have a limited mechanical force, however, these degrade rapidly. In contrast, non-natural bioinks have less biocompatibility, but these can be characterised by great printability and appropriate mechanical properties. Consequently, certain researchers apply a mixture of natural and synthetic gels, a so-called hybrid bioink exploiting their advantageous features. Bioink-selection depends on multiple factors: 1) the type of bioprinting technology, 2) the characteristics of the model tissue (e.g., stiffness, elements in the microenvironment), 3) the necessity of shape-preserving, and 4) the appropriate crosslinking process and application, the influencing effects on cell proliferation, differentiation, and survival. Crosslinking stabilises the 3D bioprinted structure, preserving the viability of the cellular elements of the bioink. This causes physical and chemical modifications in the bioink which ensures that the printed layers remain together. There are several options for crosslinking: e.g., enzymatic (fibrinogen + thrombin = fibrin); ionic (alginate—CaCl_2_); chemical (alginate—horseradish peroxidase); physical (gelatine-methacrylate—UV), or thermal (gelatine—high temperature) which can be performed either before or during or even after the printing process is finished (58). 3D bioprinting is a very effective tool, however, the standardisation of 3D bioprinting protocols is essential, and additionally, multi-faceted improvements are also required: 1) developing printing protocols, 2) standardising the materials used as bioinks, 3) creating novel biomaterials which have more sophisticated physical and biological properties, 4) improving the usability of 3D printed structures, as well as establishing test systems required for these (59, 60).

### 3D bioprinted and biofabricated cancer models

**Living cell 3D bioprinting technologies require some major tools** - An appropriate number of cells, bioinks, bioprinters, the specific printing design, and pre/post-processing before and after bioprinting. Additionally, biological tests and other biochemical, molecular biology or morphology studies also need special handling with the printed 3D materials. The development of this technology has required newly printable and biocompatible (adequate cell-compatible) materials. 3D bioprinting is easier with cancer cells in correlation with their unlimited cell growth supporting the production of a huge number of cells for bioinks. Additionally, we could combine cancer cell bioinks with cancer-associated and/or “normal” cells, and finally, the layer-by-layer bioprinted materials can form living cancer tissues during longer *in vitro* culturing.

Applying traditional 3D culturing so-called spheroid cultures, e.g., where cells are maintained in non-adherent plates or hanging drops (without applying matrix-embedded technology, only with the cells’ own matrix production) created by manual seeding of the cells, results in non-uniform cell distribution. Spheroids developed in these types of culturing methods could have various shapes, moreover, final analyses show high statistical deviation in the experimental datasets. For modelling the heterogeneity and the real *in vivo* situation of cancer tissues, new biofabricated models are required considering to achieve better complexity and standardisation. New approaches—forming small concave wells using a bioprinter, casting forms (grids) for cells or printing similar drops with bioinks (with homogenous cell concentration)—were started to be used in several tumour types (e.g., glioma, sarcoma, breast or cervical cancer cells) (61). The widespread extrusion-based and droplet bioprinting technologies provide more homogenous, controllable size, cell number and shape distributions, as well as potentially “real” tissue formations for tumour biology studies. One of the first layer-by-layer cell printing applications described better cell seeding uniformity and long-term viability (>90%, 14 days) of the printed primary cells (62). Additionally, after 2010, in some studies, magnetic levitation of the tumour cells and fibroblasts was applied aiding the formation of tumour spheres (breast cancer) with defined cellular composition and density by *Leonard’s* method (63). Others started to print 3D printed and *in vitro* cultured models with different cancer cell lines (64). In this decade, several functional tests were reconsidered, e.g., bioengineered 3D bone-mimetic structures have been started to be used for bone metastasis models (65), as well. These bone-mimetic and osteogenic niches are developing and are useful to test the effects of new compounds *in vitro* with potential inhibitory effects in breast tumour cell colonisation in the bone (66). In 2017, researchers started to fabricate multi-cellular bioprinted models for drug penetration and toxicity tests to investigate tumour tissue-like and liver tissue models (67, 68). Organovo and some other companies developed new 3D bioprinted liver tissues for drug tests (69). Several tissue-mimetic structures have been developed combining stem cells with organoid, spheroid cultures or 3D bioprinting in biofabrication. Organoid technologies have recently been exponentially developing; however, these technologies are very expensive and need a special supplement and careful handling. These technologies and different types of cancer cells (e.g., glioma, neuroblastoma, breast, kidney, colorectal, and lung cancer models) have been applied. Furthermore, rare tumours have to also be represented in these innovative approaches (e.g., chondrosarcoma, pelvic carcinomas) (70).

**FIGURE 3:**
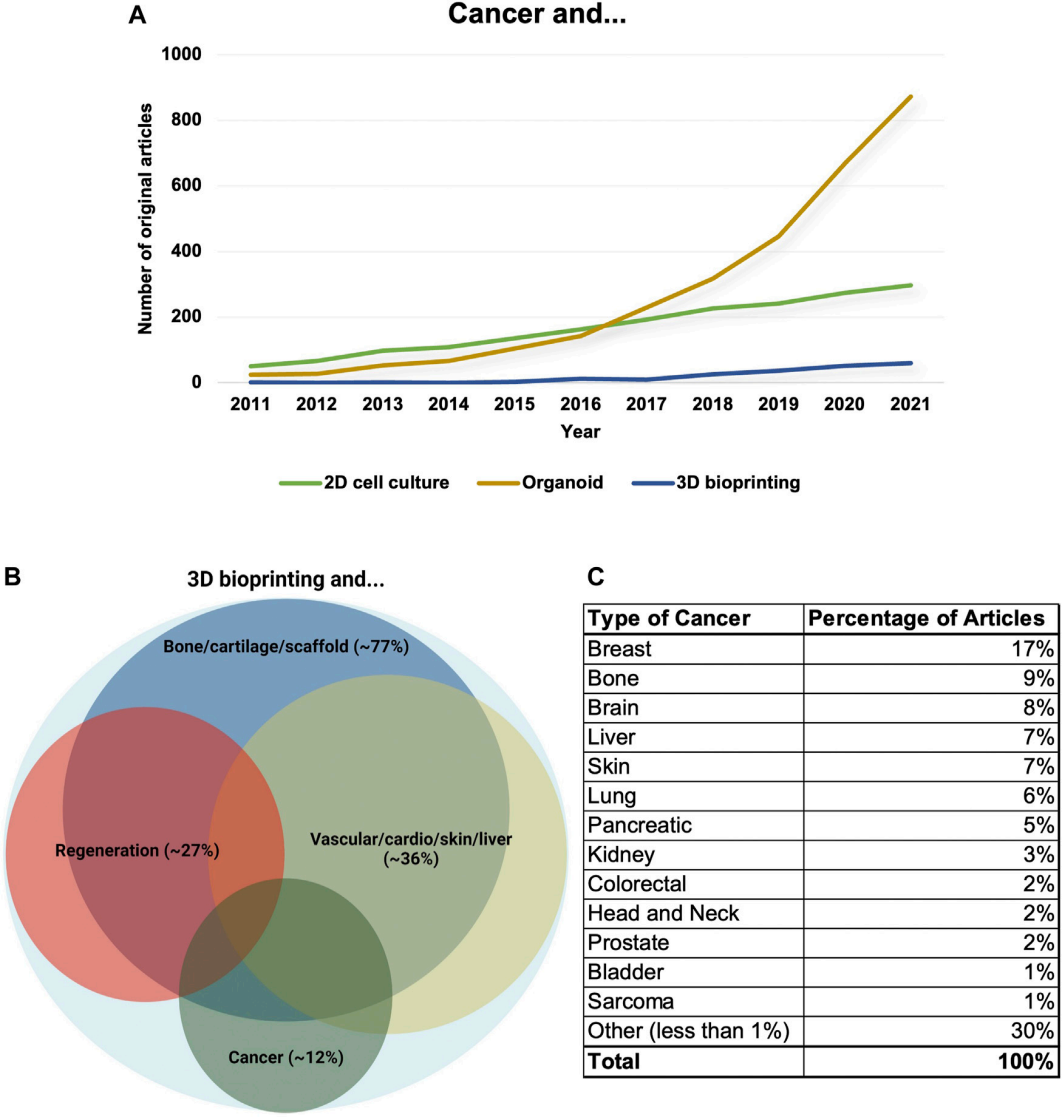
The number and distribution of cancer research papers regarding 3D bioprinting. **(A)** The number of research papers between 2011 and 2021 mentioning cancer models combined with 2D cell culture/organoid/3D bioprinting. The data show that the number of publications using 2D cell culture models is stagnating. Additionally, the organoid research area has increased faster for the last years and 3D bioprinted model systems have just been developing, but the application of 3D bioprinting technologies would also be increased rapidly in the future. **(B)** Original research papers which mention 3D bioprinting in relation with different scientific disciplines (>2260) mainly focused on 1. creating bone/cartilage and scaffold, 2. regeneration, 3. applications in the creation of vascular/cardio/skin/liver tissues/mini-organs and 4. cancer research. The distribution and the areas of the frontiers are also shown by Venn-diagram, the size of different circles represents the percentages of publications excluding review papers. **(C)** The distribution of cancer types among cancer research-related non-review and experimental papers (we excluded the papers where cancer research was only mentioned as a potential other research area or where 3D bioprinting was highlighted among technologies that are spreading or could help develop). Tumour types where the percentages of publications could not be higher than 1% merged into other studied malignancies groups.

**Analysing the increasing number of 3D bioprinting-related publications** - About 500 publications are available regarding cancer research and 3D bioprinting shows that about 1/3 of the publications are reviews or not cancer-specific papers (only mention cancer research as a potential another research area where 3D bioprinting technologies are spreading or could help develop new cancer models). Among these ∼500 publications, there are about 180 original experimental papers mentioning cancer models and 3D bioprinting. In these studies, breast- and lung cancer, and brain tumours are represented in higher numbers (∼40 breast cancers or ∼20 each of lung cancers and central nervous system malignancies), but in these studies mainly only one tumour type was used in correlation with the presented research projects until the end of 2021 (Figure 3).

**After several pioneer 3D cell culture studies, the development of new breast cancer models** - Was started to be tested, therefore, we summarise the recent history of breast cancer model development. Different 3D cultures, 3D basement membranes, and 3D hydrogel scaffolds with breast cancer cells highlighted the importance and alterations of many different behaviours (metabolic alterations, adherence, migration, sensitivity etc.) and the role of cancer microenvironments *in vitro* models and suggested engineering for 3D tumour models (71, 72). After creating manually prepared breast tumoroids in matrigel, *Swaminathan* and colleagues investigated directly bioprinted breast epithelial spheroids with different bioinks and combined these spheroids with HUVEC cells as co-cultures. They validated the remaining viability and analysed the morphology of these spheroids (73). Reid et al. described and demonstrated that using a small number (defined concentration) of tumour cells in bioprinted collagen gels, forms tumoroids and could mimic lumen formation when cancer cells are co-printed with “normal breast epithelial cells” (74). In further works, Mollica et al. tested different hydrogels and decellularised breast tissues with human breast cancer cells using 3D bioprinting and showed that using these technologies they could maintain and culture large 3D bioprinted organoids and tumoroids (75). They described the direct bioprinting technology of 3D multi-cellular breast spheroids with endothelial cells (76). In another breast cancer model work, tumour cells and adipocytes were used and monitored the viability of cells after printing in a 10-day culturing course. They described that direct printing of these co-cultures resulted in morphology, localisation, and distribution changes of cells in the printed tissue-mimetic structures (77). An important topic of the bioprinting technique is the vascularised structure development which could increase the complexity of the generated models. There are many attempts with magnetic-ring and coaxial bioprinting to build some tubular structures between spheroids covered with endothelial cells, *in vitro* forming tissues (78,79,80,81). Cancer-associated adipocytes have a special role in the tumour microenvironment, these cells are involved in some new models using co-printed, co-cultured breast cancer cells with adipocytes to increase the similarity to real tissue environment (82). Besides, there are efforts to build more complex tissue-mimetic structures with stromal cells (fibroblasts), breast cancer cells, and endothelial cells to increase the phenotypic similarities *in vitro* in 3D bioprinting protocols (83).

**The central nerve system (CNS)-derived 3D models** - Have also been developed regarding the need for new and more effective targets and therapeutic treatments. These models require the reconstitution of the complexity and heterogeneity of glioblastoma and neuroblastoma tissues, and additionally, the special tumour-stroma interactions and blood-brain barriers for accelerating potential therapeutic interventions (84). These models represent scaffold-free 3D bioprinted spheroid cultures or co-printed, co-cultured glioblastoma or other CNS malignancy-derived tumour cells (e.g., neuroblastoma) cells with the associated macrophages, stromal cells to build the special microenvironment (85,86,87,88). To develop more reliable models in these diseases, patient-derived newly isolated glioma cells are preferred instead of traditional glioma cell lines which were long cultured in 2D cultures (89), and another research direction is to combine these with 3D bioprinted blood-brain barrier models (90, 91).

There are new efforts to validate bioprinted organoids/spheroids in 96-well plates drug screening with several cancer cell lines and patient-derived tumour cells including carcinomas, glioblastomas, sarcomas, and melanomas, respectively (92, 93). Additionally, many new bone metastatic behaviour tests or cancer tissue-associated tumour antigenicity tests have been developed using 3D bioprinters and breast cancer models (94–96). These models and their applications combined with microfluidics and chips, such as tumour-on-chip, will revolutionise both drug and patient-derived tests in the near future (34,97–99).

Overall, the feasibility and complexity of biofabricating multi-cellular, cell-laden bioprinted tissue-mimetic models with the real human microenvironment of breast tumours have started to be developed in many laboratories, however, standardising these is necessary for future applications in different high-throughput drug tests and patient-derived tumour model developments (100, 101).

To complete this short review about the developing 3D bioprinted cancer models, we show our new 3D bioprinted breast cancer model and some new experiences with its establishment and suggestions for others who start applying this cutting-edge technology shortly. In our previous work, we described the metabolic characteristics and differences of the already used breast cancer models and highlighted that 3D bioprinted models are closer to the *in vivo* situation than the others. In this review, we supplement these data with the already followed long-term growing capacity and the *in situ* expression patterns and differences of some previously not studied proteins and their heterogeneity in the 3D bioprinted breast cancer model. Additionally, differences in doxorubicin and rapamycin sensitivity among 3D bioprinted, traditional 2D cell culture systems and *in vivo* xenograft models using luminal B subtype human breast cancer cells could also be highlighted.

## Materials and methods

### Cell culturing and reagents

ZR75.1 (ATCC-CRL1500), luminal B subtype human breast cancer cell line was used in our experiments. Cells were grown in 10% foetal bovine serum (FBS, Biosera), glutamine (2 mM) and gentamycin-contained RPMI-1640 media (Biosera—Nuaille, France) at standard cell culture conditions. Different treatments were applied in 96-well plates, tissue culture flasks, and 3D bioprinted tissue-mimetic scaffolds. Before the treatments, the bioprinted scaffolds were maintained for 7 days, while the cells in 2D cell cultures were incubated for 24 h. After media refreshment, the 72-h treatments were carried out in 96-well plates, tissue culture flasks, and on scaffolds (minimum 6 parallels maintained in every well of 6-well plates) for sensitivity tests. mTORC1 inhibitor rapamycin (Rapa; 50 ng/mL; Focus Biomolecules, Plymouth Meeting, PA, United States), the natural anthracycline antibiotic and chemotherapeutic agent doxorubicin (Doxo; 50 ng/mL; TEVA, Debrecen, Hungary) and their combinations were applied regarding our previous mTOR inhibitor combination sensitivity studies (102).

### 3D bioprinting

For printing, two types of bioinks were used: a. cell-containing-gel: 3% alginate and 1% gelatine (Merck-Sigma-Aldrich, Darmstadt, Germany) bioinks were mixed with cells (1 × 10^7^/mL) immediately before printing; b. cell-free-gel, more rigid gel, 6% alginate and 11% methylcellulose (Merck-Sigma-Aldrich). The scaffold layout (6 layers alternately) was designed with GeSiM Robotics software and performed by an extrusion-based bioprinter (Bioscaffolder 3.2, GeSiM, Radeberg, Germany). The printing conditions were the following: radius and height (2.5–5 mm, 0.5–1 mm); interlayer angle (90°); the distance of infill (1.5 µm); printing speed (10 mm/s); needle diameter and height (110 µm for cell-free-gel and 50–50 µm for cell-gel); pressure (400 kPa for cell-free-gel and 20 kPa for cell-gel). The scaffolds were post-processed by CaCl_2_ crosslinking (200 mM, 2 min) and washed twice then maintained in culture media (103).

### Cell viability assays

For quantitative analysis of cell viability and proliferation, Alamar Blue (AB) and Sulforhodamine B (SRB) assays were used regarding mainly to standard protocols—the 3D scaffolds were transferred into new 96-well plates (1 scaffold/well/100 µL media) directly before the measurements. The fluorescence (change) of AB (Thermo Fisher Scientific) was measured after a 4-h incubation, the detected signs were evaluated as relative fluorescence units (RFU) using a fluorimeter (570–590 nm; Labsystems International; Ascent software v. 2.6—Vantaa, Finland) culture system independently. In SRB assays, 10% trichloroacetic acid (60 min; 4°C; Merck-Sigma-Aldrich) fixation, washing steps, and overnight drying were applied before SRB (Merck-Sigma-Aldrich, 0.4 m/V % diluted in 1% acetic acid; 50 µL/well; 15 min for 2D and 1 h for 3D plate; RT) staining. The washing steps were performed carefully with 1% acetic acid then plates were left to dry overnight again (the overnight drying has special importance in the case of scaffolds). The bound SRB was re-dissolved in Tris base solution (10 mM; 150 µL/well, Merck-Sigma-Aldrich) and measured by LabSystems Multiskan RC/MS/EX Microplate Reader (570 nm; Labsystems International; Transmit Software Version 4.5—Vantaa, Finland). Relative cell proliferation was calculated in the percentage of control cells. The proliferation assays were performed with six parallels in three independent experiments.

### *In vivo* experiments

The *in vivo* experiments were performed in the Animal Care Facility unit located at the Department of Pathology and Experimental Cancer Research Institute (permission—No# PEI/001/1733-2/2015), the experimental protocols were approved by the Institutional Ethical Review Board (permission—No# PE/EA/801-7/2020). To create ZR75.1 human xenograft mouse models, 2.5 × 10^6^ cells in 100 µL RPMI-1640 media were implanted subcutaneously into the breast region of 8-week-old female SCID mice. The size of the tumours and the body weight were registered in a 3-week treatment course. Afterwards, the mice were sacrificed and tumours were removed, formalin-fixed, paraffin-embedded (FFPE) and sectioned or freshly frozen and lysed for further analyses. Tumour volume calculation was performed using the following equitation: π/6 × (2×shorter diameter + longer diameter^3^)^3^.

### Protein analysis (immunohistochemistry and WES^TM^ simple)

Immunohistochemistry was carried out using FFPE scaffolds, xenografts, and 2D cell culture slides (gel-embedded spheroids—10^5^ cells/mL) or cytospins. After deparaffinization, antigen retrieval was applied on FFPE materials (citric acid, pH 6, 30 min, pressure cooker). The fresh culture slides and cytospins were fixed in ethanol. After endogenous peroxidases and aspecific immunoreactions blocking, the used primary antibodies were the following: anti-ALDH1 (Cell Signaling; #54135; 1:200), anti-cleaved-caspase-3 (Cell Signaling; #9661; 1:1000), anti-COXIV (Cell Signaling; #4850; 1:2000), anti-LDHA (Cell Signaling; #3582; 1:400), anti-phospho-histone-H3 (Cell Signaling; #9701; 1:100). To visualize the reaction, Novolink™ Polymer Detection Systems (Leica Biosystems, Wetzlar, Germany) were used with 3,3′-diaminobenzidine (Dako, Carpinteria, CA, United States) and haematoxylin counterstaining. The stained slides were evaluated using Pannoramic Viewer Software (3D Histech). Quantitative protein expression from the cell, tissue, and scaffold lysates was investigated with WES^TM^ Simple (ProteinSimple 004-600; Minneapolis, MN, United States) a fully-automated Western blot system regarding the instructions of manufacturer using Anti-Rabbit or Anti-Mouse Detection Kits (ProteinSimple DM-001, DM-002). The samples were processed in a 12–230-kDa Separation Module (ProteinSimple SM-W004). To dissolve the 3D bioprinted scaffolds, sodium citrate was added to each sample (0.1 M, 30 min, RT), then the samples were centrifuged and lysis buffer (Tris—50 mM, pH 7.5, glycerol 10%, NaCl 150 mM, Nonidet-P40 1%, NaF 10 mM, phenylmethylsulfonyl fluoride 1 mM, Na_3_VO_4_ 0.5 mM) was added to the pellet. To evaluate protein content, Bradford reagent was used (Bio-Rad, Hercules, CA, United States). The primary antibody dilutions were 1:50 (applied primary antibodies: anti-COXIV, anti-LDHA, anti-Rictor (Cell Signaling; 2140)) and β-actin (Cell Signaling; #4970) was the normalisation control in our measurements. The electropherograms were analysed with Compass software 6.1.0 (San Jose, CA, United States). The original WES^TM^ Simple graphs were attached to Supplementary Documentation.

### Statistics

To calculate standard deviations (SD) and mean values, the results of three independent experiments with three or more parallels were evaluated. Statistical analysis was performed using PAST (version 3.24) software. Data evaluation of *in vitro* experiments was performed using Student’s t (two-tailed) test. Statistical significance was defined as *p* < 0.05.

The included figures (Figures 1–3.) were created with BioRender (https://biorender.com) covered by the Department of Pathology and Experimental Cancer Research, Semmelweis University, institutional license.

## Results and discussion

At Semmelweis University, in the Tumour biology – Tumour metabolism laboratory, different breast cancer cells (the well-known triple-negative MDA-MB-231, MDA-MB-468, luminal A—T47D or luminal B—ZR75.1 etc.) were used to construct 3D bioprinted models for performing a metabolic comparison with the same cell lines maintained in different culture systems. As we previously described, ZR75.1 luminal B breast cancer cells formed lumens after bioprinting and some-day maintenance (103). In this presented brief study, the continuous and significant growth activity of 3D bioprinted tissue-mimetic structures was detected by different proliferation tests or cell number analyses from 3 to 5 days after bioprinting to ∼3 weeks. Both the AB and SRB tests showed a fine growth curve, but around the 21st day of culturing, the detected metabolic activity and the protein content were not increased further, suggesting that the cell growth stopped. The metabolic activity changes can be followed immediately by monitoring the alterations of the pyridine nucleotide pool (nicotinamide-adenine-dinucleotides); however, the cellular protein content alters slower. Therefore, a higher amount of proteins could be detected in an extended time frame. This could be an explanation for the observed higher alteration in metabolic activity. Additionally, in the detected 21-day time period, the tumour growth of these 3D bioprinted materials was comparable with the xenotransplanted cells, where the tumour growth monitoring could be started after the tumours became palpable (1 week after inoculation) and continued for additional 3 weeks. The tumour size during this period reaches an intolerable level in SCID mice, therefore, the treatments usually need to be discontinued. These growth curves and their comparisons show that the optimal time range to perform drug tests (toxicity or proliferation tests) falls between 10 and 18days *in vitro* model systems using either 3D bioprinted materials at standard conditions or insert cultures. In *vitro* studies, we usually analyse the effects of 72-h treatments (this period is drug-dependent), which allows faster sensitivity tests than the xenograft experiments. In the 3D bioprinted *in vitro* models, we can apply these 72-h treatments after maintaining the bioprinted scaffolds for 3–10 days, but treating and culturing these printed cells can be continued even longer.

**FIGURE 4:**
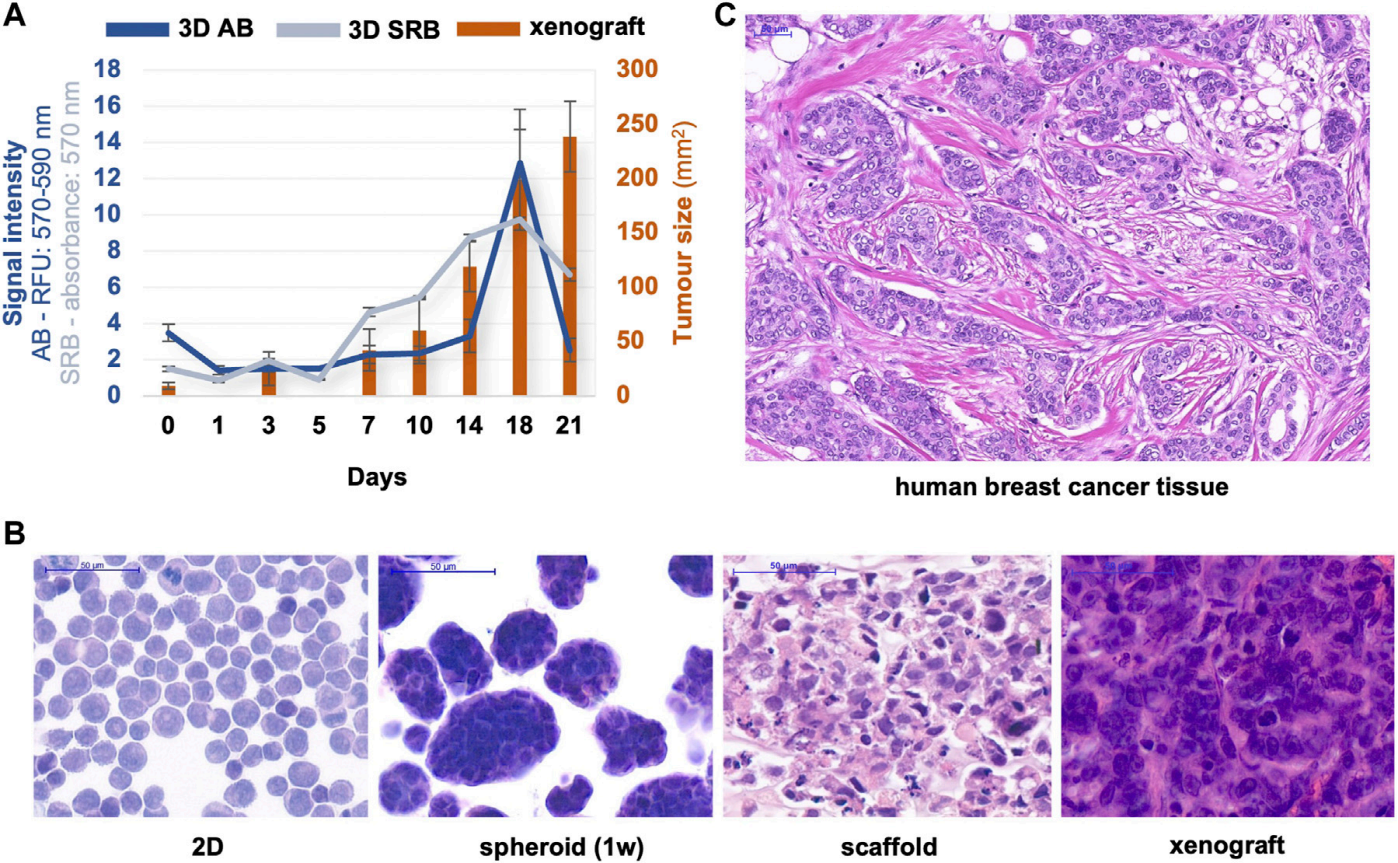
Proliferation/tumour growth and morphological characteristics of different *in vitro/in vivo* models of ZR75.1. **(A)** The growth of *in vivo* xenotransplanted and 3D bioprinted scaffold of ZR75.1 tumour. Tumour growth was indicated by the calculated tumour volume of the xenograft (right scale), while the increase in cell amount in 3D scaffolds was estimated by both Alamar Blue (AB) and Sulforhodamine B (SRB) proliferation tests (left scale). **(B,C)** Microscope images of haematoxylin-eosin-stained slides from the human xenograft mouse model, 3D bioprinted scaffold (1-week maintaining), spheroid cell culture (1-week maintaining), 2D cell culture (prepared with cytospin) of ZR75.1 cells **(B)**, human luminal B type breast cancer tissue section **(C)**. (scale bar: 50 μm).

The morphological characteristics of haematoxylin-eosin stained samples of different cell cultures, 3D printed materials, and *in vivo* xenotransplanted ZR75.1 cells were shown in Figure 4. The characteristics, the cell-cell contacts, and the lumen formation could be recognised both in xenografts and in 3D bioprinted scaffolds, and these were even more comparable with the real morphology of luminal-type breast tumours as we show in a representative human case. The heterogeneous size and shape of monomorphic cells and the cells in monolayer 2D cell cultures are much more similar to each other than to the printed and *in vivo* growing breast cancer cells (Figure 4).

**FIGURE 5:**
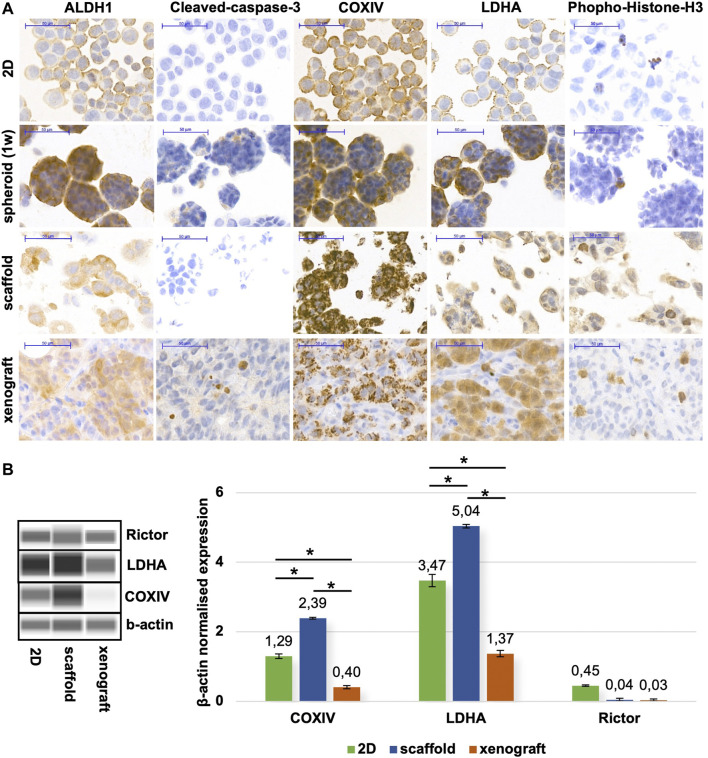
Metabolic alterations of different *in vitro/in vivo* models of ZR75.1. **(A)** Immunostainings of 2D cell cultures (prepared with cytospin technique), spheroid cell culture (maintained for 1 week), *in vivo* xenotransplanted ZR75.1 tumour, and 3D bioprinted scaffold (maintained for 1 week). The expression of ALDH1 (aldehyde dehydrogenase 1), Cleaved-caspase-3 (apoptosis marker), COXIV (Cytochrome c oxidase complex IV), LDHA (lactate dehydrogenase A), Phoshpo-Histone-H3 (mitotic marker). Immunohistochemistry was accomplished with brown (DAB, diaminobenzidine) substrate and haematoxylin counterstaining (scale bar: 50 μm). **(B)** Different maintaining condition (2D; scaffold; xenograft) affects protein expression pattern in ZR75.1 cells and xenograft tumour. WES^TM^ Simple was used to detecting metabolic enzymes (LDHA—lactate dehydrogenase A, COXIV—cytochrome c oxidase subunit 4) and Rictor expressions (left panel). Densitometric analysis was performed to present the normalised protein expression differences and was used β-actin as a loading control (right panel). *p < 0.05.

The 7/10/14/18-day cultured printed scaffolds form tissue-like structures in which the cells have a distinguished morphology compared with the 2D cultured, single cells, and even in the confluent cell cultures. To further analyse the characteristics of different model systems, immunohistochemistry stainings were performed on FFPE xenograft specimens, scaffold tissues and cytospin slides (Figure 5A). As we detected metabolic processes with COXIV, ALDH1 (oxidative phosphorylation markers) and LDHA (glycolysis marker) stainings, the tissue heterogeneity can also be represented in a 3D bioprinted scaffold as it can be in tumour xenografts and human tumours. The observed differences in heterogeneity can support the understanding of the possible adaptation mechanisms, especially in the case of altered metabolic pathway activities in tissue masses *in situ* (104). The heterogeneity of ALDH1 and COXIV stainings was increased, especially in 3D structures, which could correlate with potential metabolic rewiring processes. These findings are in association with site-dependent nutrient and oxygen distribution changes regarding the *in situ* heterogeneity in highly or less proliferative cell groups depending on the vascularisation of tumours (105). There are several publications which suggest that in starving conditions, breast cancer cells alter their glycolytic phenotype, and start to oxidase glucose on one side, if there is enough oxygen, however, use the Warburg glycolysis in oxygen-depleted or pseudo-hypoxic regions (106, 107). These heterogeneous staining patterns were detected in xenografts and 3D bioprinted materials based on the expression patterns of ALDH1, COXIV, and LDHA. In correlation with these, significantly higher growth capacity (increased proportion of phospho-histone-H3 positive cells) was also detected in these tissue-like cells and xenograft tumours vs. spheroid and 2D cell cultures. The active-caspase 3 positive apoptotic cells can also be found in the inner part of spheroids and xenografted cells. These apoptotic cells were represented at a very low level as usual (108) in 2D cell cultures (especially with a slower proliferation rate close to reaching total confluence) and in the 7-day maintained 3D bioprinted scaffolds since these “cultures” survive and proliferate at these time points. In nearly confluent 2D cell and spheroid cultures, more homogenous metabolic enzyme expressions were found, additionally, these cultures contain more uniform cell shapes and homogenous stainings. In our 3D bioprinted models, we could detect the developing resistance against many mono-treatments as we described in our previous paper (103). Formerly, we studied the *in situ* heterogeneously stained mTOR activity markers. In that study, we could compare and perform quantitative analyses including the expression differences of the above-described COXIV, LDHA, and the mTORC2 complex-specific Rictor scaffold protein expressions. These quantitative evaluations made with the use of WES^TM^ Simple and tissue lysates could not show the heterogeneity but highlighted that the 3D bioprinted models are closer to the xenograft model regarding metabolic aspects based on the expression patterns of the few studied enzymes. These confirm many previous hypotheses and findings described by other tumour models (Figure 5B).

## Future perspectives

There are several studies about the applications of 3D models in breast cancer research including 3D spheroids (hanging drops and ULA) and only a few biofabricated 3D models. In most of these studies, mainly the less aggressive MCF7 (luminal A) or the more aggressive triple-negative (MDA-MB-231) breast cancer cell lines were used. Only a few papers describe multi-cellular models, 3D models combined with vessels, adipocytes, fibroblasts or 3D bioprinted tissue mimetic *in vitro* structures in the field of breast cancer research (82, 83, 109). We also have experience printing triple-negative breast cancers using MDA-MB-23 and MDA-MD-468 cell lines. To describe and present breast cancer bioprinting in our practice, we show some new results with another breast cancer cell line, the luminal B type ZR75.1 cells. The already published printed breast cancer models have almost similar characteristics, however, different research groups use various bioinks as we analysed and referred 30 research papers. Furthermore, the bioink composition and the type of printing technologies (e.g., inkjet, extrusion-based printing) vary in these publications (Table 1).

**TABLE 1:** 3D bioprinting technology application in breast cancer studies.

		Bioink		3D technique	Cell lines	Novelty	References		
		Group	Composition						
Manually Prepared 3D Model		N + S	Matrigel + synthetic sheets	Cells were seeded on preformed multi-layered mesh sheets	MDA-MB-231	Could create different levels of hypoxia in tissue mimetic structure	Karen A. Simon et al.	2014	(110)
		N	Chitosan + hydroxyapatite	Cells were seeded on the preformed bone biomimetic scaffold	MDA-MB-231, MCF-7	A novel biomimetic engineered nano-bone model for bone metastatic studies	Wei Zhu et al.	2015	(65)
		N	Collagen-I + hyaluronan + laminin + fibronectin	Manually prepared 3D model	patient-derived breast tissue	Growing patient-derived breast cancer cells in ECM matrix	Ethan S. Sokol et al.	2016	(72)
		S	GelMa	Manually prepared microengineered tumour model	MDA-MB-231, MCF-7, MCF10A	Modelling two distinct regions of the tumour microenvironment with differential stiffness	Nitish Peela et al.	2016	(111)
		N + S	Alginate-gelatin blend, HA-PEGDA blend	Cell-containing hydrogel beads formed manually	MDA-MB-231, MCF-7	Cellular responses depend on different hydrogel properties	Rafael Schmid et al.	2020	(112)
		N	Fibrin-based	Manually prepared gel-embedded spheroids	MDA-MB-231	Vascularised metastatic breast tumour model	Madhuri Dey et al.	2021	(79)
Pre-printed cell-free mold/matrix		S	PEG-based + hydroxyapatite	3D bioprinted bone matrices, then cells were seeded on the bioprinted scaffold	MDA-MB-231, FOB	Fabricating artificial bone matrices then hosting breast cancer cells and osteoblasts for bone metastatic studies	Wei Zhu et al.	2016	(113)
		N	Porcine skin + human decellularised adipose tissue	3D preprinted mold, then cells were seeded on the bioprinted scaffold	MCF-7	Characterisation of the photothermal properties of gold nanorods with bioprinted 3D complex tissue constructs	Ki-Hwan Nam et al.	2021	(114)
		S	PEOT/PBT	3D bioprinted scaffold, then cells were seeded on the bioprinted scaffold	MDA-MB-231	Cells are more dormant in this 3D model	Afroditi Nanou et al.	2022	(115)
Live-cell 3D bioprinting	Bioprinted droplets	S	GelMa	3D bioprinted droplets	MDA-MB-231	3D tumour model chip with “layer cake” structure as an innovative 3D drug screening system	Mingjun Xie et al.	2020	(116)
		S	PEG-4MAL (+- RGD)	3D bioprinted droplets	MCF-7, MDA-MB-231	3D bioprinted model for *in situ* and real-time measurement of cell movement, migration and invasion	MoonSun Jung et al.	2022	(117)
		S	PEG-based	3D bioprinted droplets	MCF-7, MDA-MB-231, fibroblast	These 3D tissue cultures can readily be used with standard 2D high throughput assays	Martin Engel et al.	2022	(118)
	Bioprinted mono-culture scaffolds	N	Human and rat-derived hydrogels, rat-tail collagen	3D bioprinted scaffolds	MCF-7, MDA-MB-468	Generate large organoids/tumoroids for studying cell and native ECM interactions	Peter A. Mollica et al.	2019	(75)
		N	TIB - PBS used as bioink	3D bioprinted scaffolds	MCF-7	Bioprinting activates key pathways implicated in drug resistance, cell motility, proliferation, survival, and differentiation	Aleli Campbell et al.	2020	(119)
		N	Polypeptide-based	3D bioprinted scaffolds	MDA-MB-231	Breast cancer (cell line) and lung (PDX) cancer models for drug sensitivity tests	A. Gebeyehu et al.	2021	(93)
		N	alginate-gelatine, alginate-gelatine-matrigel blend	3D bioprinted scaffolds, then continuous passaging	MDA-MB-231	Harvested cells are used for continuous passaging and reprinting in 3D bioprintable alginate–gelatine systems (up to three rounds)	S. Flores-Torres et al.	2021	(120)
		N	TIB - PBS used as bioink	3D bioprinted scaffolds	MCF7, MDA-MB-231	Higher resistance in bioprinted structures	Aleli Campbell et al.	2021	(121)
		N	Tissue derived matrix, gelma, alginate, collagen-I	3D bioprinted with 3D bioplotter	MCF-7	Recreate the complex composition of breast tumours	B. Blanco-Fernandez et al.	2022	(122)
		N	Alginate-gelatine blend	3D bioprinted scaffolds	MCF-7, CD44^+^ MCF-7	Drug-resistant spheroids were able to maintain their drug-resistant phenotype during 3D culturing	Sera Hong et al.	2022	(123)
		N + S	Decellularised porcine fat + gelma	3D printed construct	MCF-7	New ECM-like hybrid bioinks were developed	You Chen et al.	2022	(124)
		N	Alginate-based	3D bioprinted scaffolds	ZR75.1	3D bioprinted cultures represent higher similarity to the *in vivo* situation	Titanilla Dankó et al.	2022	(103)
Bioprinted complex structures, co-cultures		N	Rat-tail collagen gels	3D bioprinted tumoroids	MCF-7, MDA-MB-468, MCF-12A	Bioprinted multicellular organoids to generate tumoroid arrays for assay standardisation	John A. Reid et al.	2019	(74)
		N	Alginate-gelatine blend	3D bioprinted complex tumour model	MCF-7, SKBR3, HCC1143, MDA-MB-231, HUVEC, patient-derived tissue	Generating complex tumour models - investigating cells’ maturing, self-organisation, heterogeneity, migration, therapeutic response, signalling	Ellen M. Langer et al.	2019	(83)
		N	Alginate-gelatine blend	3D bioprinted scaffolds	MCF-7, stromal cells	Modelling the breast cancer tumour environment with adipocytes and breast cancer cells	Sarah Chaji et al.	2020	(77)
		N	Matrigel, gelatine-alginate, collagen-alginate	3D bioprinted scaffolds	MDA-MB-231, MCF-7, MCF10A	Bioprinting multicellular breast tumour spheroids	Swathi Swaminathan et al.	2020	(76)
		N	Thiol-modified HA-based	3D bioprinted constructs (mono- and co-cultures)	MDA-MB-231	Generating a more complex bioprinted breast cancer model (3D adipose tissue model + 3D breast cancer model)	Hannes Horder et al.	2021	(82)
		N	Sodium-alginate	3D bioprinted droplets on mesometrium tissue	4T1	A tumour microvasculature model with cancer cells	Ariana D. Suarez-Martinez et al.	2021	(80)
		N	Matrigel	3D bioprinting with pre-formed 3D breast-epithelial spheroids and HUVEC networks	MDA-MB-231, MCF10A	Direct bioprinting of breast epithelial spheroids on pre-formed HUVEC networks to create a 3D multicellular co-culture tumour model	Swathi Swaminathan et al.	2021	(76)
		N	Collagen-based, fibrin-based	3D bioprinted complex immune-cancer model	MDA-MB-231	3D tumour models were fabricated with increased complexity to study immune-cancer interactions	Madhuri Dey et al.	2022	(109)

N, natural; S, synthetic; GelMa, gelatin methacrylate; HA, hyaluronic acid; PEGDA, poly(ethyleneglycol)diacrylate; PEOT, poly(ethyleneoxide-terephthalate); PBT, poly(butylene-terephthalate); PEG-4MAL, polyethylene-glycol-4-maleimide; RGD, arginylglycylaspartic-acid; TIB, thermal inkjet bioprinting.

Bioinks can be divided into two groups: bioinks containing only one component (natural or synthetic biomaterial) or bioinks formulated with different combinations of biomaterials. Usually, they have several components to achieve appropriate printing and cell- or biocompatibility, therefore, bioinks have several components with different features (Table 2). Alginate-based bioinks stabilised and CaCl_2_ stabilised preferably avoiding mutagenic UV for human cell printing (e.g., in the case of GELMA) (155). In the other part of these works, matrigels decellularized ECM with additional collagen or other gradients, GELMA, fibrinogen or hyaluronic acid (HA), and PEG- (polyethylene glycol) based materials are also used (82, 109, 124). The main problem with the published descriptions and protocols is that the used biomaterials are different and not fully characterised or specified in most of the papers. To start bioprinting and select the optimal conditions and bioinks is not an easy task, however, many companies provide different bioinks. Based on these, the first step, which is one of the most important ones, is the pre-processing to select the optimal bioink for the cells. Every laboratory has its way and strategy, therefore, there are no same and comparable results regarding the last few years. Hopefully, in the next years, some protocols will be cleared and standardised in 3D bioprinted cancer and breast cancer models depending on the applications (e.g., drug test, migration/metastasis/tumour progression models).

**TABLE 2:** Bioinks in cancer research.

	Base	Examples of cancer model application	Advantages	Disadvantages	Crosslinking	Examples of “ready-to-use” bioinks at companies	Ref.
Derived from natural sources	Alginate	Drug delivery model	Low cost	Poor cell adhesion	Ionic	Cellink - PhotoAlginate®-INK, CELLINK Bioink, GelMA A	(125–127)
		Cancer stem cell	Good printability	Poor stability			
		Breast cancer	High biocompatibility	Immunogenicity			
		Melanoma	Mild crosslinking conditions (Ca2+)	Non-biomimetic ECM		Growdex - GrowInk™-ALG, PhotoAlginate®	
		Tumour spheroids	Rapid gelation				
	Gelatine	Cholangiocarcinoma	Excellent biocompatibility	Low viscosity at rt or higher	Chemical		(128–130)
		Bladder cancer	Low cost	Needs temperature control	Thermal	Cellink - PhotoGel®, GelMA	
		Tumour spheroids	High cellular adhesion	Low mechanical strength (higher if blended with other bioinks)	UV	Advanced Biomatrix - GelMA, PhotoGel®, BioInx - GelMa, EASYGEL INX	
			High solubility in water		Covalent		
			Gelation is thermally reversible		Enzymatic		
	Cellulose, nanocellulose	Drug delivery model	Ecm-similarity	Low viscosity (cellulose nanocrystals)	Enzymatic	Cellink - CELLINK Bioink, GelMa C	(131–133)
		Gastric cancer	Excellent biocompatibility	Needs to be mixed with other natural biomaterials	UV	Growdex - GrowInk™-N, GrowInk™-T, GrowInk™-ALG	
		Cervical cancer					
		Pancreatic cancer					
	Matrigel	Tumour spheroids	Most used material in cancer research	Cannot be used alone (complex rheological behaviour, low mechanical properties)	Thermal		(134–136)
		Many types of cancer	Excellent biocompatibility	Limited use in vivo			
			Very well characterised for organoid/spheroid formation	Expensive High batch variability			
	Collagen-I	Tumour spheroids	Excellent biocompatibility	Low shape fidelity	pH	Cellink - Lifeink® Collagen Bioink, Advanced Biomatrix - PureCol® EZ Gel, RatCol®, Lifeink®, PhotoCol®, CollPlant - Collink.3D	(137–139)
		Neuroblastoma	High cellular adhesion		Thermal		
		Breast cancer	Low immunogenicity				
			Excellent printability			
			Enzymatically degradable			
			Mechanical and structural properties close to native tissue				
	Hyaluronic-acid	Tumour spheroids	Excellent biocompatibility	Poor mechanical strength	Physical or covalent	Cellink - PhotoHA®, Advanced Biomatrix - PhotoHA®	(140, 141)
		Melanoma	Highly tunable (wide variety and high degree of potential chemical modifications)	Mainly used as a mixture			
		Breast cancer	Interact with cell receptors				
			Fast gelation				
			Promotes cell proliferation				
	Agarose	Leukaemia	Good biocompatibility	Poor cell viability if not blended with another biomaterial	Thermal		(142)
			High ECM-similarity	Poor printability (needs high temperature for dispensing - 70°C)	Ionic		
			Thermo reversible (non-toxic) gelling	Poor cell adhesion			
			High stability	Not degradable			
	Fibrin	Drug release model	High shape fidelity (depending on fibrinogen-thrombin concentration)	Medium cell adhesion	Enzymatic (fibrinogen - thrombin)	Cellink - CELLINK FIBRIN	(143, 144)
		Glioblastoma	Excellent biocompatibility	Low mechanical properties and limited printability			
			Enzymatically degradable				
			Rapid gelation				
	Polypeptides	Ovarian cancer	Self-assembly	Low cell viability (low ph)	Ionic-complementary	Manchester Biogel TheWell Bioscience - VitroINK	(145, 146)
			Adapted for soft-tissue applications and in conjunction with other materials				
	Decellularised matrix (dECM)	Many tumour models depending on dECM	Renders natural ECM	Low stability	Depends on chemical modifications		(147–149)
			Tissue specific	Protein denaturation during fabrication processes			
			High biological relevance	Poor printability if not mixed with another biomaterial			
			High cell survival	Long procedure		
				Undefined and inconsistent		
				Loss of native ECM			
Derived from synthetic sources	Acrylamide	Melanoma	Wide-range of elasticity and flexibility	Needs other supportive material for cell proliferation (alginate, gelatine, etc)	UV	Cellink - PhotoAlginate®, PhotoGel®, PhotoHA®, GelMA	(150–152)
		Breast cancer	Most standardised protocol			Advanced Biomatrix - Mebiol®, PhotoGel®, PhotoHA®, PhotoCol®, PhotoAlginate®, PhotoDextran®, BioInx - GelMa	
	PCL		Good mechanical strength and rigidity	Not compatible with live-cell bioprinting (mainly used as a preprinted frame or mold)	Depends on the natural biomaterial used	Cellink - CELLINK PCL	(153)
			Controllable degradation	Needs other supportive material for cell proliferation (alginate, gelatine, etc)			
	Pluronic	Vascularised tissues, complicated tissue constructs	High shape fidelity	Short of cell-binding domains	Covalent	Cellink - Pluronics 40%, Advanced Biomatrix - Pluronics 40%	(154)
		Drug release model	Good printability	Low cell viability			
				Poor mechanical strength			

Due to the exponential developments and cutting-edge technological solutions, it is expected that several biological mechanisms would be characterised better and model systems would become more standardised in the case of tumours and other disease models. This improvement is greatly supported by the fact that not only the EU, EMA, and FDA but several other authorities also urge the implementation of the 3R strategy in various investigational studies including the basic and applied research areas, respectively. This initiates the replacement of animal models with *in vitro* ones, of which industrial application could only be realised if standardised models would be used or available in the future.

The wide variety of bioinks, bioink protocols as well as innovative bioprinters and technologies point out that various factors have to be taken into account during project planning, not even mentioning the measurements and settings required in certain experiments, the design and usability of assays suitable for monitoring growth and proliferation changes, or other biological and molecular processes (56). As we described earlier, the gel composition is diverging nearly in all laboratories (or within the same research group based on the researcher’s choice). Based on our previous experiments, which are not presented here, and others’ results, it can be stated that the composition of bioinks and gels used for printing can significantly change not only the growth and survival of cells but also the morphological features of cells that form the spheroids and tissue-like structures. Thus, the printing conditions and the exact composition of materials have special importance so that the published results could be reproduced, at least in case of using the same cell lines. Potentially, the most important point will be to examine how drug preselection tests performed with specific 3D bioprinted model systems show and predict the results previously obtained in the phase trials and the future outcomes. If it can be proved that with the use of these 3D bioprinted or the developing organoid models and their combination such *in vitro* tissue, organ models could be created which would be more appropriate for performing more efficient drug screening tests than in animal models *in vivo*. Then, there would be the time for tightening the regulations. Accordingly, most of the studies would be carried out with human cell-based *in vitro* tests to replace several animal experiments. All of these can lead to the point that the pharmaceutical developments mentioned in the introduction will be more successful, efficient, and consequently cost-effective in the near future.

## Data Availability

The original contributions presented in the study are included in the article/Supplementary Material, further inquiries can be directed to the corresponding author.

## Supplementary material

SUPPLEMENTARY FIGURE S1 The original and unadjusted images of WES^TM^ Simple electropherograms.
