# Supplementary figures and images for: 3D bioprinting and the revolution in experimental cancer model systems—A review of developing new models and experiences with in vitro 3D bioprinted breast cancer tissue-mimetic structures
Source: Pathol Oncol Res. 2023 Feb 9;29:1610996. doi: 10.3389/pore.2023.1610996 (PMC9946983; doi:10.3389/pore.2023.1610996)

## Slide 1
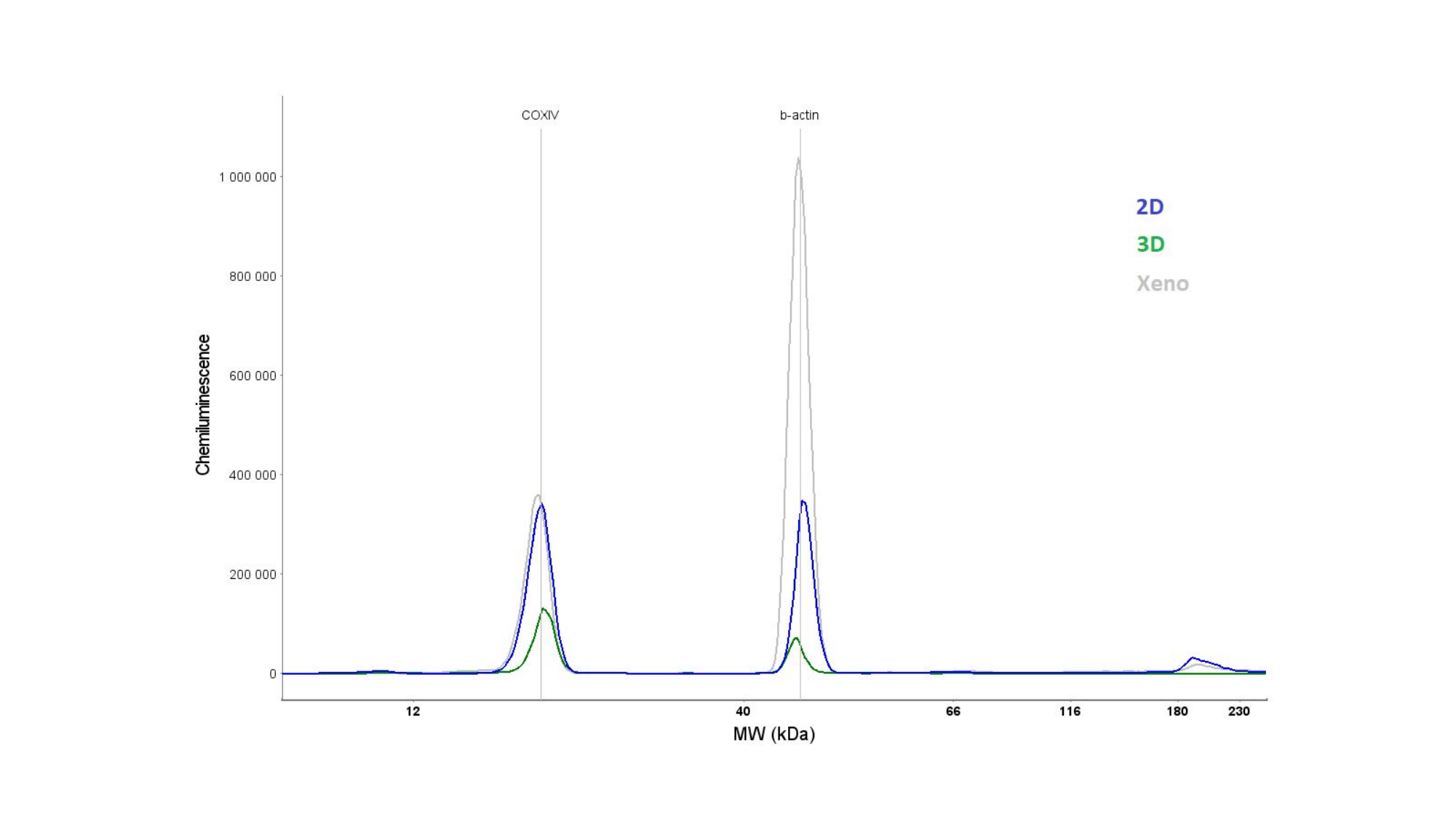

## Slide 2
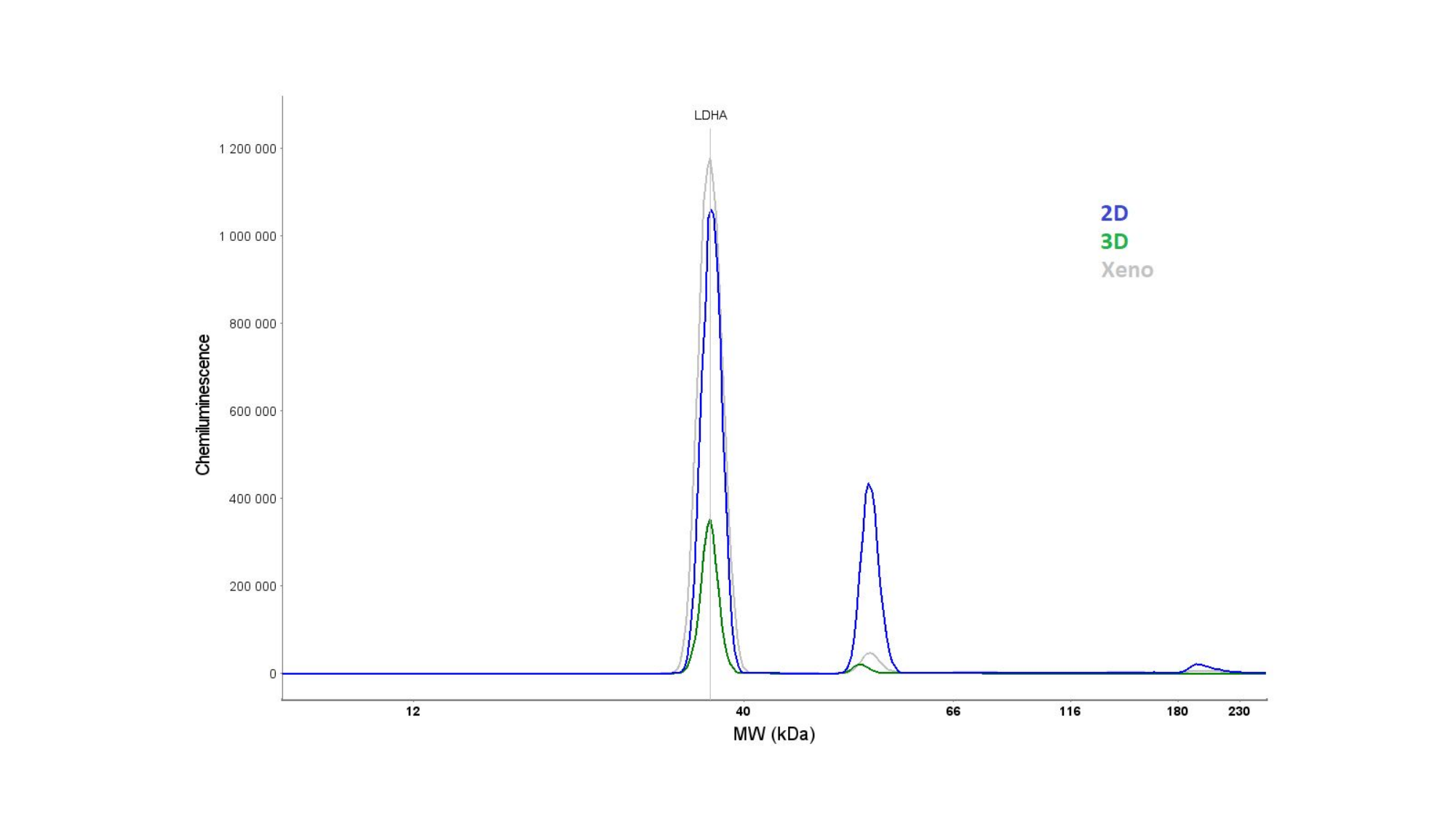

## Slide 3
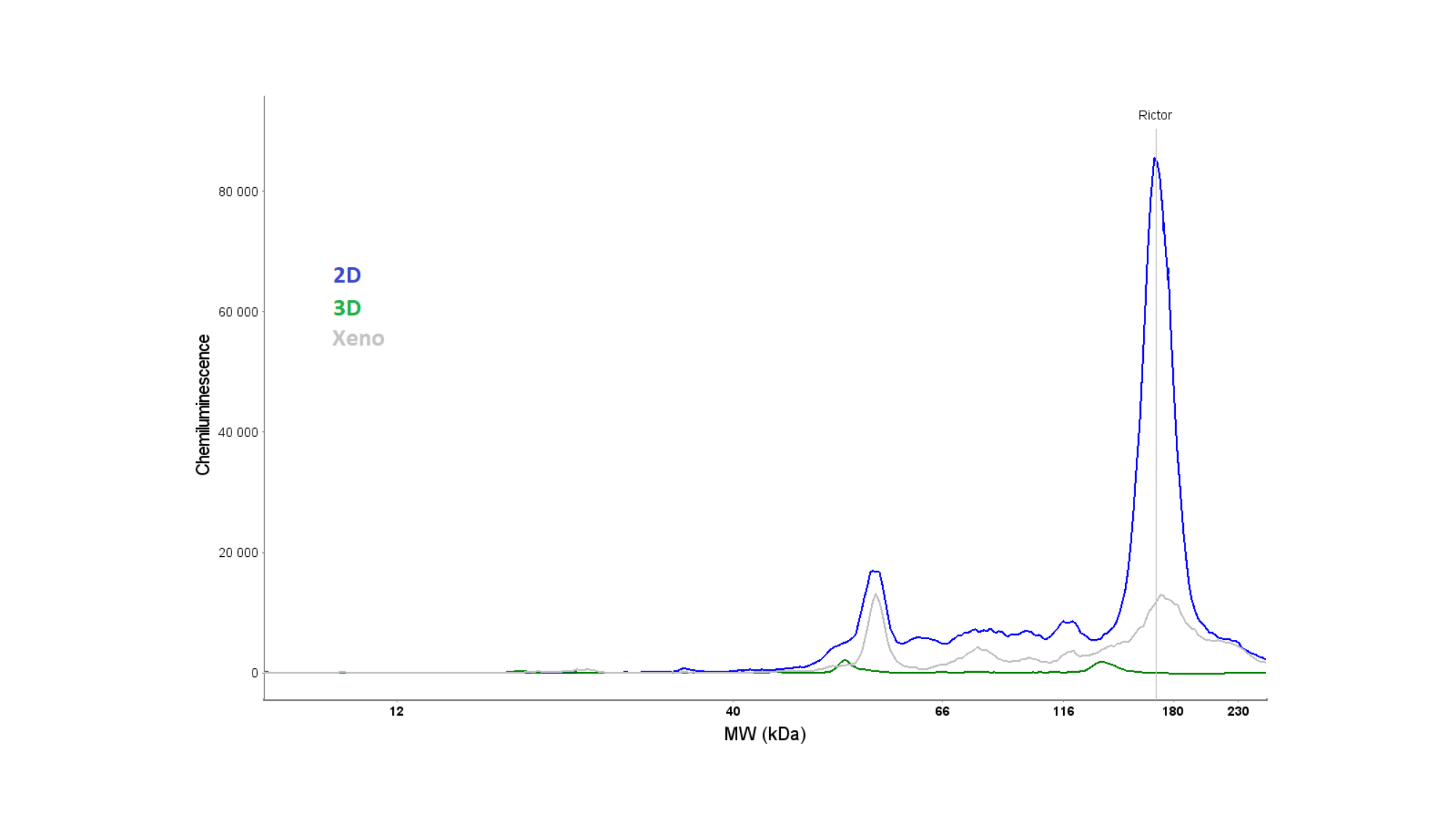

Supplement: Supplementary file 1 [file Presentation1.PPTX]
